# Protective effect of α-lipoic acid against radiation-induced fibrosis in mice

**DOI:** 10.18632/oncotarget.6952

**Published:** 2016-01-20

**Authors:** Seung-Hee Ryu, Eun-Young Park, Sungmin Kwak, Seung-Ho Heo, Je-Won Ryu, Jin-hong Park, Kyung-Chul Choi, Sang-wook Lee

**Affiliations:** ^1^ Department of Radiation Oncology, University of Ulsan College of Medicine, Asan Medical Center, Seoul, Korea; ^2^ Institute for Life Sciences, Asan Medical Center, Seoul, Korea; ^3^ Department of Biomedical Sciences and Department of Pharmacology, Cell Dysfunction Research Center (CDRC), University of Ulsan College of Medicine, Seoul, Korea; ^4^ Cell Dysfunction Research Center and Bio-Medical Institute of Technology (BMIT), University of Ulsan College of Medicine, Seoul, Korea

**Keywords:** radiation-induced fibrosis, α-lipoic acid, acetylation, NF-κB, PAI–1

## Abstract

Radiation-induced fibrosis (RIF) is one of the most common late complications of radiation therapy. We found that α-lipoic acid (α-LA) effectively prevents RIF. In RIF a mouse model, leg contracture assay was used to test the *in vivo* efficacy of α-LA. α-LA suppressed the expression of pro-fibrotic genes after irradiation, both *in vivo* and *in vitro*, and inhibited the up-regulation of TGF-β1-mediated p300/CBP activity. Thus, α-LA prevents radiation-induced fibrosis (RIF) by inhibiting the transcriptional activity of NF-κB through inhibition of histone acetyltransferase activity. α-LA is a new therapeutic methods that can be used in the prevention-treatment of RIF.

## INTRODUCTION

Radiotherapy (RT) is a major cancer therapeutic modality. More than 40% of cancer patients receive RT. Even when RT is curative, long-term survivors suffer normal tissue complications. High-dose radiation induces cellular damage and increases cytokine and growth factor-mediated signaling, leading to dysfunctional repair and fibrosis. Current aggressive treatment approaches, such as intensive RT or RT combined with chemotherapy, increase the risk of acute and late treatment-related complications. Late side-effects after RT in long-term cancer survivors have become an important issue [1]. Radiation-induced fibrosis (RIF), one of the most common late complications of high-dose RT [2] along with necrosis, develops within months or years of RT [1, 3]. RIF depends on the total radiation dose, fraction size, and whether RT is combined with chemotherapy or surgery. Despite advances in radiation treatment techniques, RIF remains a limiting factor of success in RT. Although decreasing the total radiation dose or irradiated volumes is the best method to prevent RIF, this could lead to a decreased therapeutic efficacy. The pathogenesis of RIF is caused by chronic damage to normal tissues by reactive oxygen species (ROS). Therefore, methods that can reduce ROS production after RT may ameliorate RIF. In fact, several antioxidants have shown protective effect against RIF preclinically and clinically [4–6]. Unfortunately, there is no clinically proven effective treatment for RIF.

Radiation-induced fibrosis (RIF) is characterized by excessive accumulation of extracellular matrix in skin and soft tissue, and the proliferation of fibroblasts is one of the most common late complications of radiation therapy. Also, RIF is an irreversible process to dead fibrous tissue and a dynamic process related to the remodeling of scar tissue by reactivated myofibroblasts.

TGF-β1 regulates the expression of genes in many biological events, such as tissue remodeling, cell proliferation, tumor development, progression, and apoptosis [7]. A key role of TGF-β1 was identified in the response to injury and in the pathogenesis of fibrosis in the lung and the liver [8–11]. Mechanistically, TGF-β1 activates receptors to induce the phosphorylation of Smad2 and Smad3. The Smad complexes then translocate to the nucleus, where they recruit transcriptional regulators, such as histone acetyltransferase (p300/CBP) and histone deacetylases (HDACs), to regulate the transcription of target genes. Recent reports revealed that TGF-β1 signaling is directly involved in the activation of p300/CBP [12], however, the mechanisms by which the TGF-β1-p300/CBP network is regulated in skin RIF remain unknown.

NF-κB is a ubiquitously expressed protein involved in inflammatory and immune responses and in cellular proliferation [13]. NF-κB is composed of a heterodimer of p50–p65 subunits [14]. p65 (RelA) is also phosphorylated or acetylated by cytokines [15, 16]. Thus, the acetylation of p65 controls the NF-κB transcriptional response and is involved in diverse diseases, such as chronic inflammation and asthma, and in the migration and resistance of cancer cells [17, 18]. Therefore, the acetylation of p65 is an attractive target for pharmaceutical development in normal soft tissue fibrosis.

The screening of natural compounds to identify histone acetyltransferase (HAT) inhibitors led to the discovery of garcinol, curcumin, anacardic acid, and EGCG, which may have value in the prevention of cancer, inflammation, and fibrosis [19, 20]. Previous results from our laboratory showed that EGCG inhibits p300 and CBP *in vitro* and *in vivo*, and that anacardic acid and curcumin inhibit Tip60, p300, and PCAF [21, 22]. p300/CBP is the global target of HAT inhibitors. Since activation of p300/CBP was reported in colorectal, breast, and prostate cancer, this signal activation may be directly associated with tumorigenesis [23, 24], however, it remains unclear whether inactivation of p300/CBP by HAT inhibitors may be suppress the proliferation and migration of normal or cancer cells.

α-Lipoic acid (LA: 5–1, 2-dithiolan-3-yl) pentanoic acid) is first isolated from bovine liver in 1951 [8] and a naturally occurring dithiol compound synthesized in the mitochondrion from octanoic acid [25]. αLA can serve as strong antioxidants and could be a potential agent in prevention of different disease that may be related to an imbalance of cellular oxidative status [11]. For instance, αLA has been used safely for more than 30 years in Germany to treat diabetes and polyneuropathies [26]. However, there are limited studies focusing on the anti-fibrotic effects and underlying molecular mechanisms of αLA [12, 13, 25]. Antioxidant activity of αLA provides therapeutic benefit in a rat model of CCl_4_-induced liver fibrosis [12]. αLA inhibits thioacetamide (TAA)-induced liver fibrosis in rats by inhibiting TGF-β1 and the MAP kinase signaling pathway [25] and inhibits bile duct ligation (BDL)-induced hepatic fibrosis in mice by inhibiting TGF-β signaling pathway [13]. In addition, αLA inhibits TNF-α-induced NF-κB activation in human aortic endothelial cells [26]. NF-κB binds to the promoter region of the pro-fibrotic makers, PAI-1 and MMP-9 and pro-fibrotic marker expression is increased by NF-κB [27]. Here we report that aLA prevents radiation-induced fibrosis in mice by inhibiting the transcriptional activity of NF-κB, especially through the inhibition of TGF- β1-mediated histone acetyltransferase (HAT) activation.

## RESULTS

### α-LA prevents development of RIF *in vivo*

**Figure 1:**
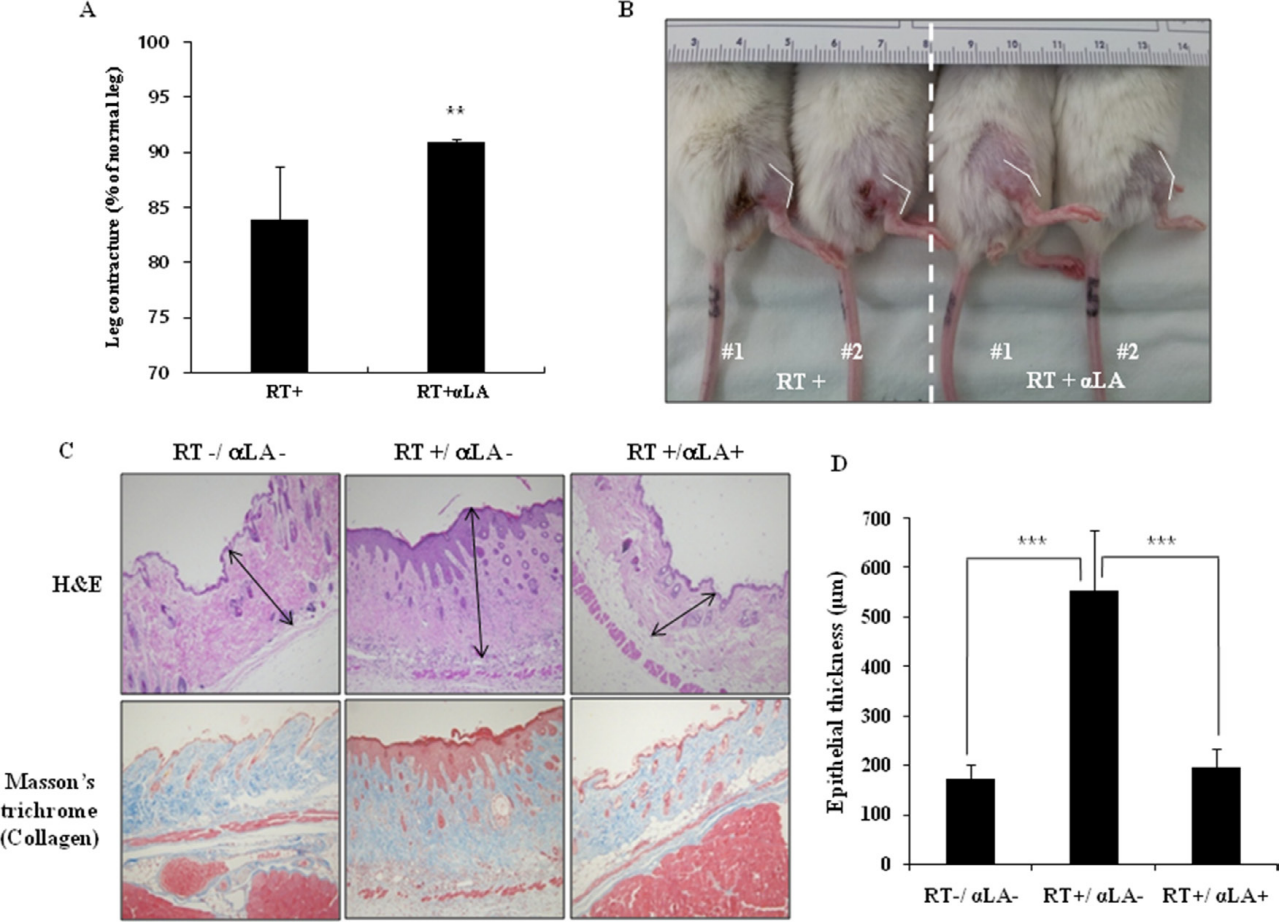
α-LA inhibits radiation-induced fibrosis in mice model (**A**) The leg contraction assay in mouse RIF model for 14 weeks after radiation. The results are shown as mean of percentages ± S.D. (**p < 0.05.) (**B**) The leg contracture of irradiated mice was dramatically improved by treatment of α-LA. (**C**) α-LA inhibits the increased epithelial thickness and collagen accumulation by radiation. The mouse skin tissue was stained by H & E and Masson's trichrome. (**D**) Epithelial thickness by α-LA treatment group (RT+/α-LA- and RT+/α-LA+).

To investigate whether α-LA blocks RIF *in vivo*, the RIF mouse model was used. As shown in Figure 1A, we performed the leg contraction assay in RIF mice. Mice were separated into three groups: one group was not exposed to radiation, a second group was exposed to radiation, and a third group was exposed to radiation and treated with α-LA. The leg contracture assay was performed 14 weeks after irradiation with 22 Gy (two times). Radiation increased leg contractures, but the leg contracture of irradiated mice was dramatically improved by α-LA. The length of irradiated legs was significantly longer in α-LA-treated mice than in control mice post-irradiation (Figure 1B).

To demonstrate the anti-fibrotic effect of α-LA in the skin and soft tissue of irradiated legs, we measured epithelial thickness from the surface of the epidermis to the base of the dermis using H & E staining. As shown in Figure 1C, the epithelial thickness was greater in irradiated leg tissues than in normal tissues and α-LA-treated tissues. α-LA reduced RIF in soft tissues. We next evaluated the ability of α-LA to protect from RIF. Collagen is a key marker of fibrotic disease. As shown in Figure 1C and 1D, radiation increased collagen expression and epithelial thickness. Conversely, α-LA lowered collagen expression and epithelial thickness in radiation-exposed legs. Collectively, these results establish that α-LA prevents the development of RIF in skin and soft tissue by inhibiting the excessive type I collagen accumulation and fibrotic response.

### α-LA represses the expression of pro-fibrotic markers induced by radiation *in vivo* and *in vitro*

According to the correlation of α-LA and TGF-β1, α-LA inhibits TAA-induced liver fibrosis in rats and BDL-induced hepatic fibrosis in mice by inhibiting TGF-β1 signaling pathway. Thus, we next examined expression of fibrosis markers, including TGF-β1, PAI-1, α-SMA, MMP2, and MMP9, in the leg tissue by immunohistochemistry (IHC). RIF was accompanied by increased expression of fibrosis markers, whereas α-LA inhibited them.

**Figure 2:**
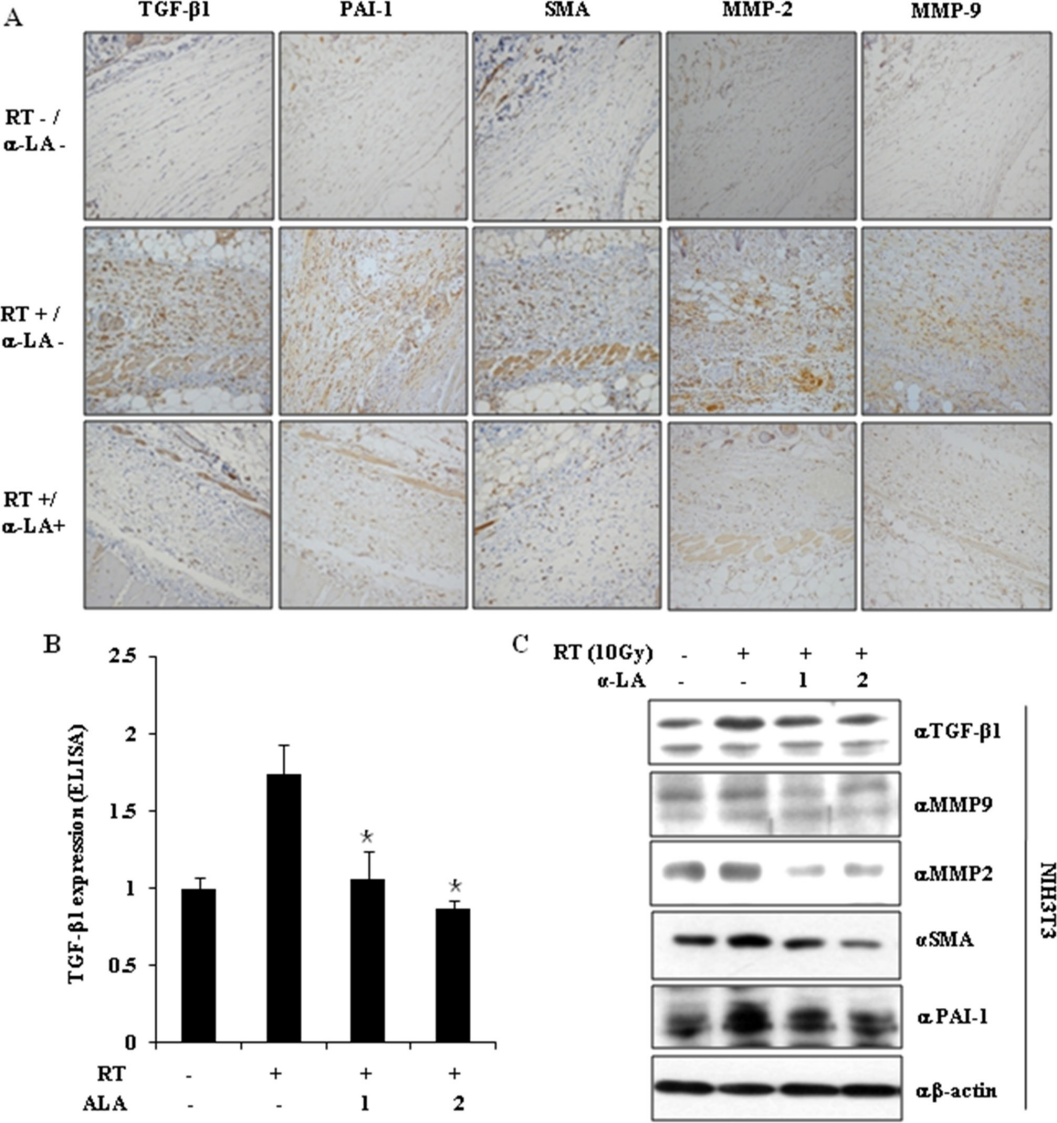
α-LA represses the activation of TGF-β1 and pro-fibrotic markers by radiation, *in vivo* and *in vitro* (**A**) α-LA inhibits the expression of TGF-β1-mediated fibrotic markers in the mouse RIF model. The mouse skin tissue was analyzed by immunohistochemistry (IHC) using the indicated antibodies. (**B**) α-LA blocked radiation-induced TGF-β1 expression, which was measured by TGF-β1 ELISA assay kits. The results were shown as mean ± S.D. calculated from three independent experiments. *p < 0.05. (**C**) α-LA attenuates the expression of pro-fibrotic markers in the mouse fibroblast NIH-3T3 cells. Cell lysates were analyzed by immunoblotting using the indicated antibodies.

Since TGF-β1 is known to be an important factor for fibrotic responses, we next examined whether α-LA inhibits the increase in TGF-β1 expression and secretion in mouse NIH-3T3 fibroblasts upon irradiation (10 Gy). As shown in Figure 2A and 2B, radiation induced TGF-β1 expression and α-LA blocked the increasing TGF-β1 expression. Western blot analysis showed that α-LA repressed radiation-induced TGF-β1, MMP-2, MMP9, PAI-1, and α-SMA expression (Figure 2C).

### α-LA directly inhibits HAT activity

RT induces DNA double-strand breaks (DSBs) and cell death. The induction of dynamic changes in chromatin structure after irradiation leads to chromatin remodeling [28]. Post-translational histone modifications play critical roles in chromatin remodeling after radiation. HATs catalyze the acetylation of histone and non-histone proteins, a process required for chromatin remodeling by radiation.

**Figure 3:**
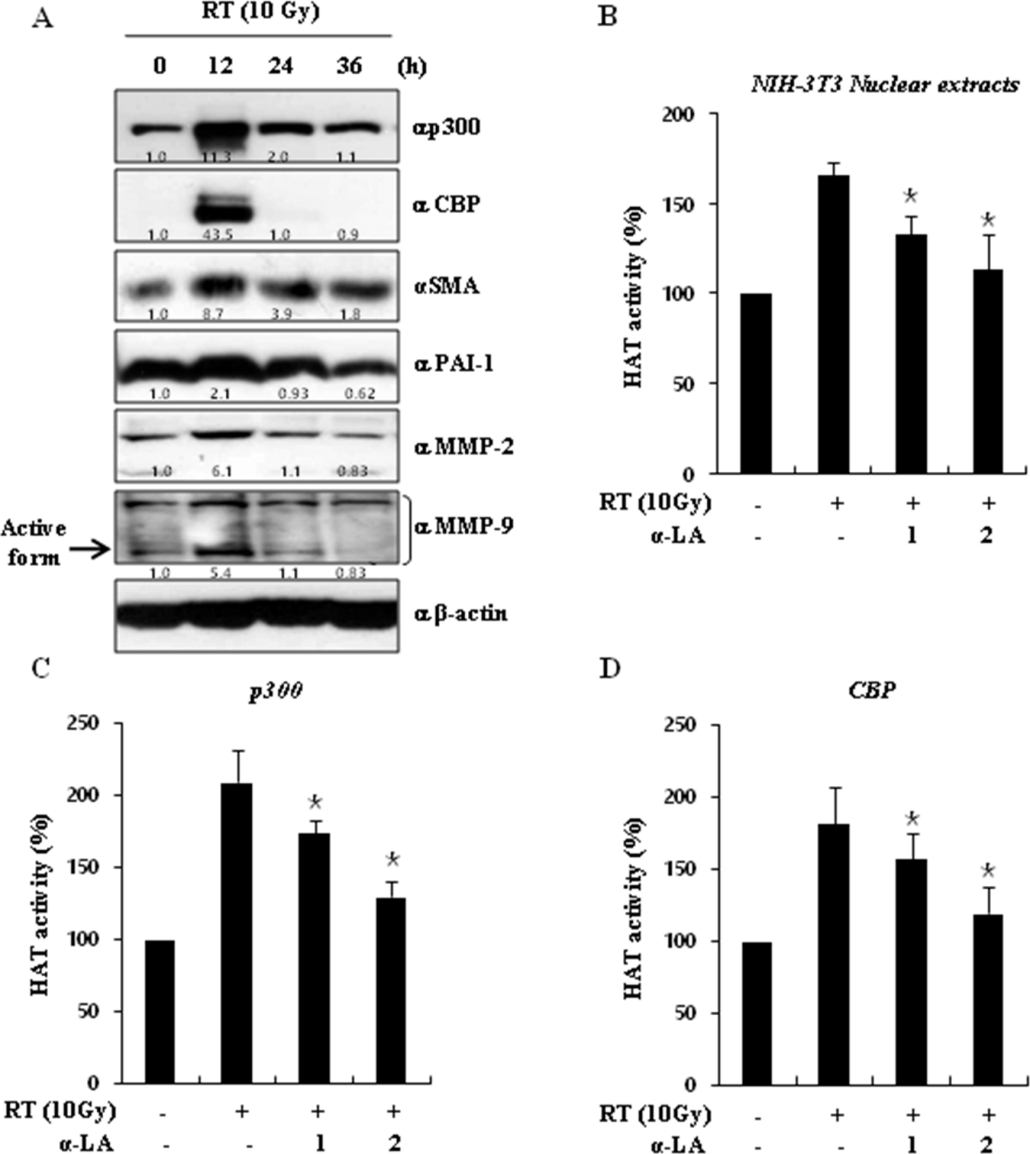
α-LA decreases histone acetyltransferase (HAT) activity (**A**) Radiation dramatically increased the expression of HAT proteins. Cells was cultured for indicated times after irradiation. The expression of p300/CBP proteins and pro-fibrotic markers were measured by immunoblotting using the indicated antibodies. (**B**) α-LA suppressed the radiation-induced activation of HATs. NIH-3T3 nuclear lysates was extracted by the cytoplasm/nuclear fraction buffers. Nuclear lysates were measured by the histone acetyltransferase assay kits. (**C–D**) α-LA prevents the activation of p300 and CBP.p300 and CBP in the NIH-3T3 nuclear lysates were precipitated by each antibody. Precipitated samples were measured by the histone acetyltransferase assay kits. The results are shown as mean ± S.D. calculated from three independent experiments. *p < 0.01.

To explore the mechanisms underlying the activation of HATs by radiation, we examined the expression of p300/CBP proteins and pro-fibrotic markers over time after a radiation dose of 10 Gy (Supplementary Figure 1). As shown in Figure 3A, radiation significantly increased p300/CBP and pro-fibrotic markers, such as MMP2, MMP9, SMA, and PAI-1, at 12 h. Further, radiation increased p300/CBP activity and α-LA antagonized HAT activity in a concentration-dependent manner (Figure 3B). Measuring HAT activity by using immunoprecipitated p300 and CBP proteins, we also showed that α-LA suppresses acetyltransferase activity (Figure 3C and 3D).

### α-LA antagonizes p300-mediated p65 acetylation

**Figure 4:**
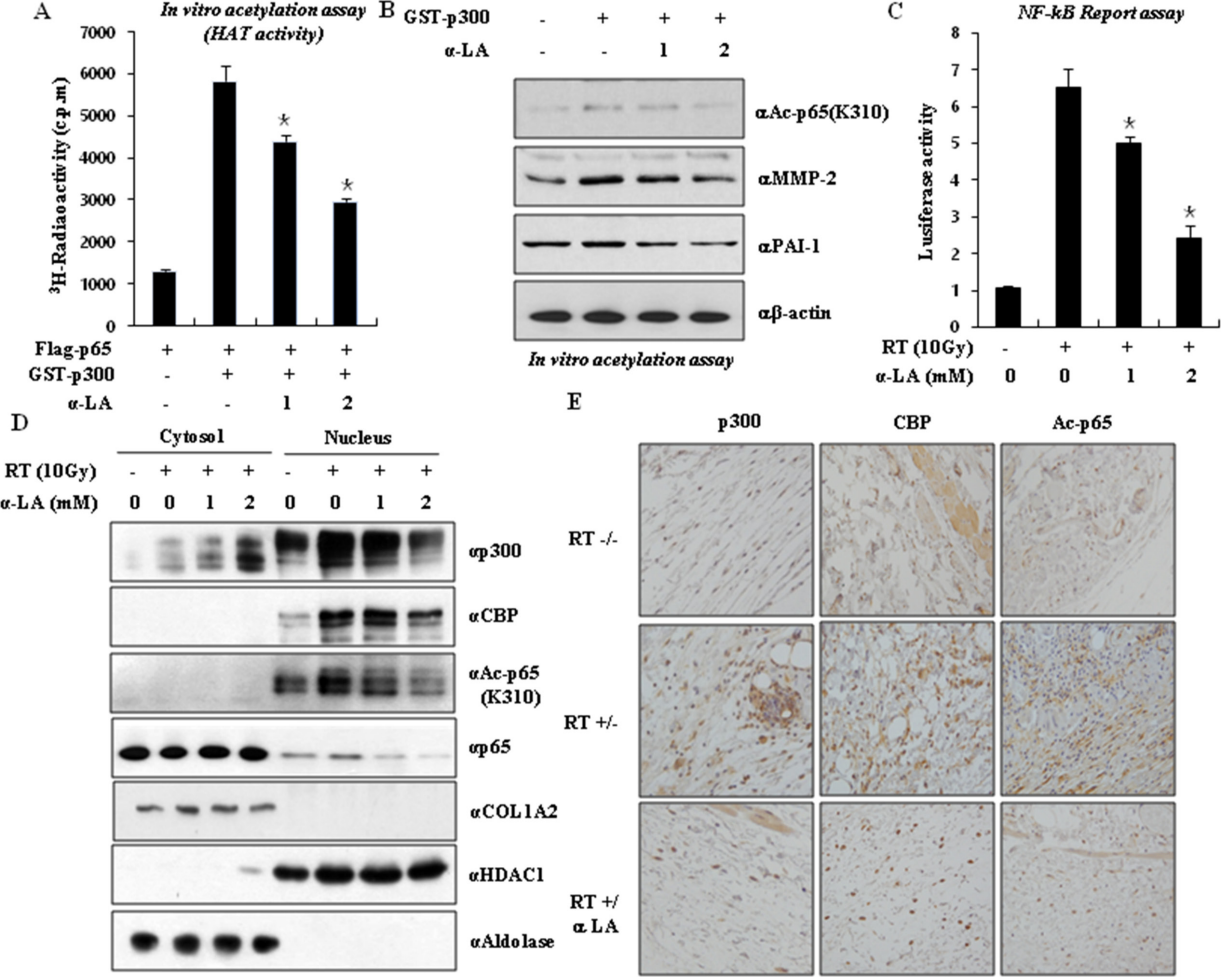
α-LA antagonizes p300-mediated p65 acetylation (**A**) α-LA induced the hypoacetylation of p65 via inhibition the HAT activity of p300 in a concentration-dependent manner. HAT activity was performed by *in vitro* acetylation assays with α-LA. Recombinant GST-p300 was incubated with Flag-p65 in the presence or absence of α-LA and samples were counted with a multipurpose scintillation counter, LS 6500 (Beckman). The results are shown as mean ± S.D. calculated from three independent experiments. *p < 0.05. (**B**) Acetylated p65 was inhibited by α-LA. p65 acetylation was detected by immunoblotting using antibody against acetylated lysine (310) p65. (**C**) α-LA inhibits the radiation-induced NF-κB activity. (**D**) α-LA treatment prevented radiation-induced p65 acetylation and translocation. NIH-3T3 cells were exposed by radiation, and then treated with or without α-LA. Cells were extracted by the cytoplasm/nuclear fraction buffers, and the fractionated samples were measured by immunoblotting using the indicated antibodies. (**E**) α-LA blocks the translocation of acetylated p65, p300 and CBP in the nucleus. The pathological relevance of acetylated p65 was observed by immunohistochemistry using the indicated antibodies in the mouse RIF tissues.

Previous results from our laboratory demonstrated that p300-mediated p65 acetylation is important for the maintenance of NF-κB function [29, 30]. Since p65 is acetylated by p300, we examined whether α-LA directly inhibits p300-mediated p65 acetylation. *In vitro* acetylation assays were performed in the presence or absence of α-LA using purified recombinant active p300 and Flag-M2-immunoprecipitated p65 as a substrate. As shown in Figure 4A, α-LA significantly inhibited the HAT activity of p300 in a concentration-dependent manner and induced the hypoacetylation of p65. These results establish that p300-induced acetylation is inactivated by α-LA and confirm that α-LA prevents p65 acetylation *in vitro*. To further demonstrate the prevention of p65 acetylation by α-LA, we assessed the effect of α-LA on p300-induced p65 acetylation *in vitro* by western blot analysis. As shown in Figure 4B, acetylated p65 was detected in the presence of purified active p300. In the presence of α-LA, p300-induced p65-acetylation was reduced, and the expression of MMP-2 and PAI-1 was inhibited. Also, NF-κB transcription activity was decreased by α-LA during RIF (Figure 4C). Taken together, we conclude that α-LA suppresses p65 hyperacetylation *in vitro* by blocking the HAT activity of p300/CBP.

To confirm the inhibition of p300-mediated p65 acetylation by α-LA, we assessed the effect of α-LA on the radiation-induced acetylation of p65 in NIH-3T3 fibroblasts. Irradiated NIH-3T3 cells were incubated with or without α-LA and analyzed using antibodies against acetylated p65 (K310), p65, p300, and CBP by Western blot. As shown in Figure 4D, the expression of p300 and CBP was increased in the nucleus, and the acetylated form of p65 was observed in the nucleus (Supplementary Figure 2). By contrast, α-LA inhibited radiation-induced p65 acetylation. Since our data suggest a critical role for α-LA in RIF development *in vitro* and *in vivo*, we sought to demonstrate the pathological relevance of acetylated p65 by p300/CBP and the therapeutic efficacy of α-LA in the RIF mouse model by immunohistochemistry. As shown in Figure 4E, α-LA dramatically suppressed the expression of p300/CBP and acetylated p65 in RIF leg tissues compared to untreated irradiated tissues. These data demonstrate the pathological relevance of acetyl-p65 and therapeutic relevance of the inhibition of p300/CBP by α-LA in the progression of RIF.

### α-LA prevents RIF by antagonizing the hyperacetylation of p65

Fibrosis is defined as a fibroproliferative process or abnormal fibroblast activation. PAI-1 is the main physiological inhibitor of fibrinolysis [31–33]. PAI-1 increases the activity of urokinase plasminogen activator (uPA) and MMP proteolytic activity in normal physiologic conditions, and maintains tissue homeostasis. Increased PAI-1 expression inhibits uPA and MMP activity, and helps wound healing. Namely, PAI-1 is significantly elevated in fibrotic tissues, and lack of PAI-1 protects different organs from fibrosis. In addition, PAI-1 directly increases ECM accumulation and indirectly inhibits matrix components by activating MMPs [34–36]. PAI-1 plays a pivotal role in the development of hepatic fibrosis [37] and is important in the fibrosis of several organs including heart, lung, kidney, liver, and skin.

**Figure 5:**
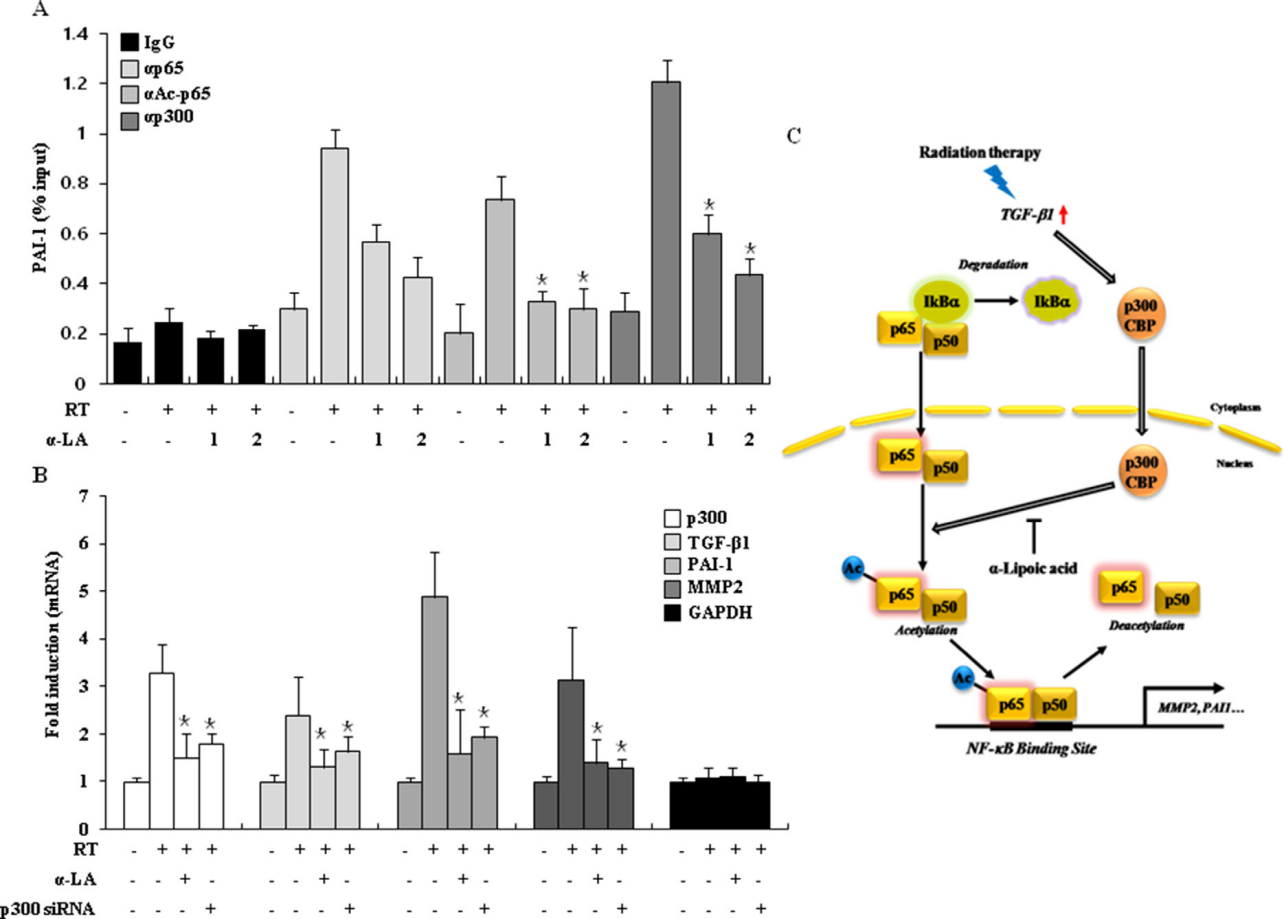
α-LA prevents RIF by antagonizing the hyperacetylation of p65 (**A**) α-LA induced p65 hypoacetylation in the PAI-1 promoter region. NIH-3T3 cells were irradiated by radiation, and then were treated with or without α-LA for various doses as indicated. ChIP assay was performed with the indicated antibodies. DNA samples were analyzed by quantitative PCR. The results are shown as mean ± S.D. calculated from three independent experiments. *p < 0.01. (**B**) The treatment of p300 siRNA or α-LA efficiently suppressed the expression of pro-fibrotic markers. Cells were exposed by radiation, and then treated with α-LA or transfected with p300 siRNA. The expression of indicated genes was analyzed by quantitative PCR. The results are shown as mean ± S.D. calculated from three independent experiments. *p < 0.01. (**C**) Model of our finding. In radiation treated cells, HAT proteins were activated by radiation-induced TGF-β1, and acetylated p65 enhances the expression of pro-fibrotic marker genes. Importantly, α-LA antagonizes acetylation of p65 and represses fibrosis-marker expression during radiation-induced fibrosis. Consequently, α-LA prevents the RIF via the inhibition of HAT activity.

To investigate the role of acetylation in p65 recruitment to the PAI-1 promoter during RIF, we conducted chromatin immunoprecipitation (ChIP) analysis at the PAI-1 promoter in irradiated cells. The PAI-1 promoter contains a well-characterized NF-κB (p65) binding site. Figure 5A shows that radiation increased the recruitment of p65, Ac-p65, and p300 to the promoter of the PAI-1 gene; however, this increased recruitment of Ac-p65 and p300 was inhibited by α-LA in a concentration-dependent manner. In addition, we tested the transcriptional repression of pro-fibrotic target genes by α-LA in irradiated cells by knocking down p300, and performed RT-PCR and Western blot. As shown in Figure 5B, α-LA efficiently suppressed the expression of pro-fibrotic markers. Most importantly, NIH-3T3 cells transfected with p300 siRNA showed similar transcriptional activation of pro-fibrotic genes as control cells. Thus, p300-mediated p65 acetylation is required for the p65-mediated transcriptional activation of radiation-induced pro-fibrotic genes. Taken together, the data strongly suggest that α-LA inhibits the hyperacetylation of p65 by p300/CBP and plays an important role as a histone acetyltransferase inhibitor during RIF (Figure 5C).

## DISCUSSION

Radiation therapy induces wound repair and, thus, the accumulation of extracellular matrix, leading to excessive fibrosis. In this study, we found that α-LA has a therapeutic effect on the radiation-induced soft tissue fibrotic change. The efficacy of α-LA in the RIF mouse model was confirmed by physical and biological approaches. The leg contracture assay showed a protective effect of α-LA on 14 weeks after radiation. We also demonstrated that α-LA inhibited the RIF in the mouse model. According to the previous literature, Type I collagen, a product of two genes, COL1A1 and COL1A2, is the major extracellular matrix component [38]. The accumulation of collagen after radiation was reduced in the RIF mouse model upon α-LA treatment. It is well known severity of RIF that is a one of the dose limiting late complications after RT. Radiation induces inflammation following wound healing process and, thus, the uncontrolled accumulation of extracellular matrix occurs, leading to excessive fibrosis. RIF is not the result of a short-term alteration in the normal repair mechanism but a dynamic process of inflammation. Several treatment methods including corticosteroids, interferon, clodronate, pentoxifylline, hyperbaric oxygen therapy, and antioxidant have been proposed to antagonize RIF [1, 39]. Despite reports of the anti-fibrotic activity of RIF antagonists, mechanisms of their action remain unclear [39]. Unfortunately, there is no standard drug or treatment method RIF for clinical practice.

The present study showed α-LA inhibited RIF and the expression of pro-fibrotic genes via inhibition of p65 acetylation. In particular, p65 acetylation correlates with the expression of pro-inflammatory and pro-fibrotic genes during fibrosis, inflammation, and cancer through the MAPK, NF-*κ*B, and STAT pathways [33]. Therefore, acetylation of p65 increases the p65 DNA-binding affinity and transcriptional activation in human diseases [40]. NF-*κ*B is activated in an acetylation-dependent manner by cytokines and growth factors. TGF-β1 induces NF-*κ*B activation and the NF-*κ*B-dependent transcription of TNF-α and IL-1β. Unbalanced NF-*κ*B expression and modification causes inflammation and cancers in humans. Furthermore, α-LA inhibited the transcription of radiation fibrosis-inducing genes. Various pro-fibrotic genes possess a NF-*κ*B subunit p65-responsive element increased by NF-*κ*B signaling. Among the known post-translational modifications of p65, acetylation plays a critical role in regulating various biological functions of NF-*κ*B including DNA binding, transcriptional activation [16].

In our study, α-LA inhibits p300/CBP activity in mouse fibroblasts. α-LA reduced radiation-induced skin fibrosis and inhibited HAT activity of p300 that occurs through increased TGF-β1 expression in NIH-3T3 cells after radiation. Ectopic expression of p300 enhances Smad-dependent collagen gene expression in normal skin and lung fibroblasts [41]. By contrast, depletion of endogenous p300 causes down-regulation of TGF-β1-mediated pro-fibrotic responses in normal fibroblasts [42]. TGF-β1 increases NF-*κ*B activation via p300-dependent p65 acetylation and the p65-dependent transcription of inflammatory mediators [43]. Although there are many candidate HDAC inhibitors, relatively little is known about HAT inhibitors [44]. Inhibitors of HDACs and HATs have been developed for several therapeutic purposes. Recently, several naturally occurring HAT inhibitors (anacardic acid, garcinol, EGCG, and curcumin) were identified and characterized by many researchers [20, 21]. Thus, we investigated the RIF prevention effect of several natural compounds with multiple HAT-inhibition activity such as EGCG, curcumin, and anacardic acid. In this study, we found that α-LA inhibited RIF in mouse soft tissues and normal fibroblasts [45].

This study demonstrated that p300 and p65 are important components of the regulation of TGF-β1-mediated transcriptional activation in RIF. TGF-β1 is the master molecule in tissue fibrosis induced by various types of injury including radiation injury [46]. Radiation can directly activate the TGF-β signaling pathway and this is a major signaling pathway in RIF. TGF-β signaling is a central mechanism in fibrotic change and, therefore, blocking TGF-β1 signaling is a reasonable strategy for protecting against RIF. The biological activity of TGF-β1 is regulated by diverse mechanisms and depends on transcriptional cofactors and the transcription potential of Smad2 and Smad3 [28]. In our previous results establishing the RIF mouse model, TGF-β1 mRNA was significantly increased in tissues of irradiated mouse 28 days after irradiation, and this could be detected for up to 3 months post-irradiation [47]. TGF-β1-mediated pro-fibrotic gene expression was suppressed by α-LA in mouse fibroblasts through inhibition of histone acetyltransferase, p300, and CBP [13]. The fibrosis related genes expressions by TGF-β1 are increased by p300. p300 is a transcriptional coactivator with acetyltransferase activity. p300 regulates the expression of various genes in specific signaling pathways involving cellular proliferation, apoptosis, and embryogenesis. Recent reports implicate p300 in the regulation of collagen gene expression by TGF-β1 [48]. The expression of p300 and CBP is significantly elevated in the skin of patients with systemic sclerosis [32, 36]. In addition, p300 levels in explanted normal fibroblasts were dramatically increased by TGF-β1, and histone H4 hyperacetylation was increased upon p300 accumulation at the collagen gene promoter [31]. Importantly, pharmacological inhibition of p300 acetyltransferase activity repressed the transcription of TGF-β1-induced pro-fibrotic genes in normal skin. Therefore, we suggest that p300 plays a critical role in the progression of fibrosis and cancer, and that its expression level may be a suitable biomarker of these processes.

PAI-1 expression was significantly elevated by radiation and α-LA inhibited the radiation-induced increased expression of PAI-1 as well as collagen, α-SMA, and TGF-β1. PAI-1 promotes normal tissue fibrosis by inhibiting ECM degradation. Since TGF-β1 increases PAI-1 at the transcription level, these results indicated that α-LA inhibits TGF-β1-induced PAI-1 expression through inhibition of TGF-β1 expression [30]. In addition, TGF-β1 increases p300 recruitment at the PAI-1 promoter [49]. p300 co-localized with acetylated p65 at the PAI-1 promoter leads to the activation of PAI-1 expression and ECM accumulation [50]. p300 plays an important role in extracellular matrix (ECM) remodeling and fibrosis in fibroblast biology [33]. In particular, the acetylation of p65 at Lys310 by p300 and CBP has an essential role in the increase in RIF, and transactivates pro-fibrotic responsive genes such as PAI-1 and α-SMA. Taken together with our data α-LA inhibited the radiation-induced HAT activity of p300 and CBP, it is suggest that suppressed these HAT activity by α-LA caused regulation of transcriptional co-activators involved in the activation of the PAI-1 promoter by p65.

In conclusion, p65 acetylation plays an essential role in RIF, and inhibition of p300 and CBP activity by α-LA inhibits TGF-β1 expression in fibroblasts. Moreover, we demonstrated that α-LA antagonizes radiation-induced p65 acetylation in mouse soft tissues. Our results demonstrate a novel protective effect of α-LA against RIF *in vivo* and *in vitro*, and identify α-LA as a novel HAT activity inhibitor.

## METHODS

### Cell culture and reagents

All cell lines were obtained from the American Type Culture Collection. NIH-3T3 cells were cultured in DMEM supplemented with 10% fetal bovine serum, 1% antibiotics, and antimycotics (Hyclone, Logan, UT,). Cells were treated with α-LA and exposed to radiation. Cell lines were propagated at 37°C in 5% CO_2_. HAT activity was measured using a colorimetric assay from BioVision Biotechnology. The antibodies were purchased from Santa Cruz Biotechnology, Upstate Biotechnology, and Cell Signaling Technology. The Lipofectamine 2000 transfection reagent was purchased from Invitrogen.

### Western blot analysis

NIH-3T3 cells were pretreated with α-LA for 2 hours and then irradiated with 10 Gy using a linear accelerator (Varian, Palo Alto, CA). Cells were incubated for 12 hours and then analyzed by Western blot. The total cellular protein content was extracted with lysis buffer (50 mmol/L Tris-Cl (pH 7.5), 150 mmol/L NaCl, 1% NP40, 10 mmol/L NaF, 10 mmol/L sodium pyrophosphate, and protease inhibitors). Proteins were separated on a 10% SDS-PAGE gel, transferred to nitrocellulose membrane, and subjected to immunoblot analysis. Results were visualized by autoradiography. Protein expression was detected with anti-TGF-β1, anti-MMP9, anti-MMP2, anti-αSMA, and anti-PAI-1 (Santa Cruz Biotechnology, Cell Signaling Technology, Danvers, MA).

### RIF mouse model

Male BALB/c mice (Central Lab. Animal, Seoul, Korea) were used for the RIF mouse model [51]. All experiments were approved by the Institutional Animal Care and Use Committee (IACUC) of the Asan Institute for Life Science, Korea. Under anesthesia, the right hind limb of each mouse received radiation doses of 44 Gy (22 Gy × 2 times for 2 weeks) using a linear accelerator. Specially designed shielding was used to protect the rest of the body, and a 1 cm thick bolus was applied over the skin to ensure an adequate radiation dose on the surface of the hind leg. After irradiation, mice were randomly divided into two groups. Each group was treated once daily (5 times/week) with saline (*n* = 8; control group) or α-LA (*n* = 8; 600 mg/kg/day; DalimBioTech, Seoul, Korea) administered orally. Mice (*n* = 4) who received no irradiation or drug were used as a negative control group. All treatments started before irradiation and continued for 14 weeks.

### Leg contracture assay

At 14 weeks after irradiation, the leg contracture assay was performed, using a specially designed Lucite jig modified from one described by Stone *et al.* [52], as described by Ishii *et al*. [53]. Briefly, an anesthetized mouse was placed in a Lucite jig, and the length of the extended leg was measured with a ruler inlaid within the base of the jig. The degree of contraction was recorded as the length of the irradiated leg and compared to that of the un-irradiated contralateral leg. Results were expressed as percentages.

### Histological and immunohistochemical analysis

Mice were sacrificed for histopathologic evaluation 14 weeks after irradiation. The skin and soft tissue of the irradiated leg was formalin-fixed, paraffin-embedded, cut into 4 μm sections, and stained with hematoxylin-eosin using standard procedures. To detect collagen accumulation in fibrotic tissues, Masson's trichrome staining was performed according to the manufacturer's protocol (Diagnostic BioSystems, Pleasanton, CA). For the immunohistochemical analysis, deparaffinized slides were incubated with anti-TGF-β1 (Cell Signaling), anti-PAI-1 (Santa Cruz Biotechnology), anti-SMA (abcam), anti-MMP2 (Santa Cruz Biotechnology), anti-MMP9 (Santa Cruz Biotechnology), anti-p300 (Santa Cruz Biotechnology), anti-CBP (Santa Cruz Biotechnology), and anti-acetylated p65 (Cell Signaling) primary antibodies, and subsequently with horseradish peroxidase-conjugated secondary antibodies. The DAB^+^ chromogen were used for signal detection (Dako Real Envision detection kit; Dako, Glostrup, Denmark). Representative images of the brown staining within fibrotic regions were captured and evaluated.

### ChIP assay and real-time PCR analysis

Chromatin was isolated as described previously [54]. Briefly, ~2 × 10^9^ NIH-3T3 cells in a 100 mm^2^ dish were treated with PBS containing 1% formaldehyde for 10 min, washed twice with PBS, and incubated with 100 mmol/L Tris (pH 9.4) and 10 mmol/L DTT at 30°C for 15 min. The cells were then rinsed twice with PBS and resuspended in 600 μL Sol A buffer [10 mmol/L HEPES (pH 7.9), 0.5% NP40, 1.5 mmol/L MgCl_2_, 10 mmol/L KCl, and 0.5 mmol/L DTT]. After the sample was centrifuged for 5 min at 500 × g at 4°C, the pellets were resuspended in Sol B [20 mmol/L HEPES (pH 7.9), 25% glycerol, 0.5% NP40, 0.42 mol/L NaCl, 1.5 mmol/L MgCl_2_, and 0.2 mmol/L EDTA] containing protease inhibitors and vigorously triturated in order to extract nuclear proteins. After centrifugation at 6, 000 × g for 30 min at 4°C, the nuclear pellets were resuspended in immunoprecipitation buffer [1% Triton X-100, 2 mmol/L EDTA, 20 mmol/L Tris/HCl (pH 8.0), 150 mmol/L NaCl, and protease inhibitors] and sonicated to break chromatin into fragments with an average length of 0.5 to 1 Kb. ChIP assays were performed with the indicated antibodies essentially as described previously but with SDS-free buffers. The antibodies against acetylated p65, p65, and HDAC3 were purchased from Santa Cruz Biotechnology, and those against RNA polymerase II and p300 were purchased from Upstate Biotechnology. The primers used for the ChIP assays included the following: mPAI-1, F, 5′-TGCTCAAGTGCTGAGTCACT-3′, and R, 5′-AGACTCATGGGAAAATCCCA-3′. The primers used for the real-time PCR included the following: mp300, F, 5′-CAGATTCCACCACAACCCCA-3′, and R, 5′-ACT AGATGGCTG AGCTGCTG-3′; mTGF-β1, F, 5′-GCTCT TGCCCTCTACAACCA-3′, and R, 5′-GTTGGACAACTG CTCCACCT-3′; mPAI1, F, 5′-GGAAGAAGACCCGATC AACA-3′, and R, 5′-GCCACGAGAATCAAATCCAT-3′; mMMP2, F, 5′-GAAACCGTGGATGATGCTTT-3′, and R, 5′-CCATCAGCGTTCCCATACTT-3′; GAPDH, F, 5′-TGATGACATCAAGAAGGTGGTGAA G-3′, and R, 5′-TCCTTGGAGGCCATGTAGGCCAT-3′.

### TGF-β1 ELISA assay

The expression of TGF-β1 was measured using Human/Mouse TGF-β1 ELISA Ready-SET-Go! kits (eBioscience, USA) according to the manufacturer's instructions. Briefly, NIH-3T3 cells were cultured by complete media and the culture media was concentrated by Centricon (Millipore, USA), concentrated culture media were plated in 96-well coated-plates with TGF-β1 antibody. Substrate solutions were added to each well. Then, the plate was incubated in R.T. for 15 min. After stop solution was added, the plate was measured using a SpectraMAX 250 Optima plate reader at 450 nm (Molecular Device Co., Sunnyvale, CA).

### Report assays

To measure NF-kB transcriptional activity, NIH-3T3 cells were transiently cotransfected with reporter construct pNF-kB-Luc and pSV40 plasmids. The Renilla luciferase reporter plasmid was included as an internal control. Cells were harvested and extracted, and the samples were measured, according to the manufacturer's instruction (Promega, USA). All reporter activities were normalized relative to Renilla luciferase activities and are presented as the means (± SD) of three independent experiments.

### *In vitro* HAT activity assay

NIH-3T3 cell nuclear extracts were prepared as described previously [22]. For HAT activity assays, immunoprecipitations were performed using anti-p300 and anti-CBP (Santa Cruz Biotechnology). HAT activity was measured by manufacturer's protocol (BioVision Biotechnology). As shown in Figure 3, to demonstrate HAT inhibition by α-LA, a Flag-p65 plasmid was transfected into NIH-3T3 cells, after which the cells were incubated for 48 h. For HAT activity assays, immunoprecipitation of p65 protein from NIH-3T3 nuclear extracts was performed using Flag M2 beads (Sigma) for 12 h at 4°C. The immunoprecipitates were collected and washed in HAT assay buffer [50 mmol/L Tris (pH 8.0), 10% glycerol, and 0.1 mmol/L EDTA]. The p300 active domain (amino acids 1066–1707) was cloned and purified as a GST fusion protein. HAT activity assays were performed using purified p300. Flag-p65 immunoprecipitates and purified GST-p300 were incubated in HAT assay buffer [50 mmol/L HEPES (pH 8.0), 10% glycerol, 1 mmol/L DTT, 1 mmol/L phenylmethylsulfonyl fluoride, 10 mmol/L sodium butyrate, 1 μL [^3^H] acetyl-CoA] with or without α-LA at 30°C for 1 h. Measurements were made in a LS 6500 multipurpose scintillation counter (Beckman).

### Statistical analysis

All values are presented as means ± standard errors. Significance was analyzed using the Student's *t*-test with the SPSS program. *P* < 0.05 was considered to indicate a significant difference.

## SUPPLEMENTARY MATERIALS FIGURES


