# Supplementary material for: Protective effect of α-lipoic acid against radiation-induced fibrosis in mice
Source: Oncotarget. 2016 Jan 20;7(13):15554–65. doi: 10.18632/oncotarget.6952 (PMC4941260; doi:10.18632/oncotarget.6952)
Supplement: Supplementary file 1 [file oncotarget-07-15554-s001.pdf]

Protective effect of α-lipoic acid against radiation-induced fibrosis in mice

Supplementary Materials

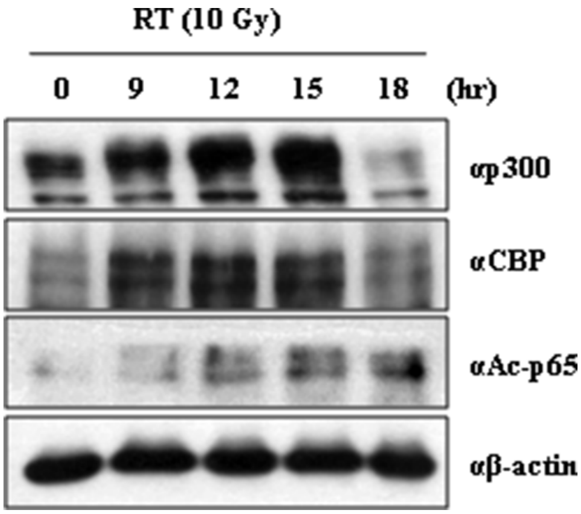

Supplementary Figure S1: Radiation induces the hyperacetylation of p65. The activated p300/CBP by radiation enhanced the p65 acetylation in radiation-induced fibrosis. Cells were exposed by radiation (10 Gy). Sample lysates were measured by immunoblotting using the indicated antibodies.

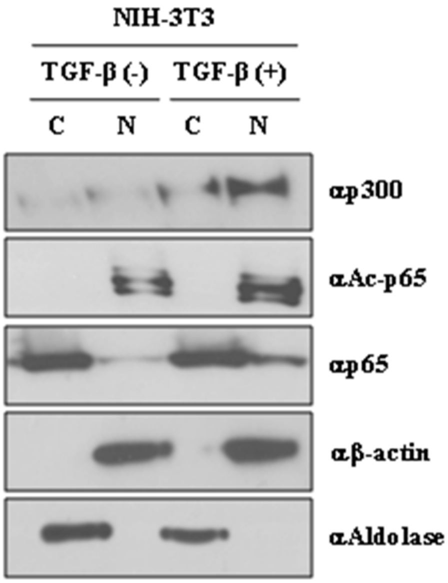

Supplementary Figure S2: TGF-β1 induces the nuclear translocation of p300 and acetylated p65. NIH-3T3 cells were treated by TGF-β1 for 12 h, and cells were extracted by the cytoplasm/nuclear fraction buffers, and the fractionated samples were measured by immunoblotting using the indicated antibodies.
